# Unmasking population undercounts, health inequities, and health service access barriers across Indigenous populations in urban Ontario

**DOI:** 10.17269/s41997-024-00957-8

**Published:** 2024-10-30

**Authors:** Marcie Snyder, Stephanie McConkey, Raman Brar, Judy Anilniliak, Cheryllee Bourgeois, Brian Dokis, Michael Hardy, Serena Joseph, Amanda Kilabuk, Jo-Ann Mattina, Constance McKnight, Janet Smylie

**Affiliations:** 1 Well Living House, St. Michael’s Hospital, Unity Health Toronto, Toronto, ON Canada; 2 Dalla Lana School of Public Health and Department of Family & Community Medicine, University of Toronto, Toronto, ON Canada; 3 Tungasuvvingat Inuit, Ottawa, ON Canada; 4 Seventh Generation Midwives Toronto, Toronto, ON Canada; 5 Southwest Ontario Aboriginal Health Access Centre, London, ON Canada; 6 Anishnawbe Mushkiki Aboriginal Health Access Centre, Thunder Bay, ON Canada; 7 Waasegiizhig Nanaandawe’iyewigamig Aboriginal Health Access Centre, Kenora, ON Canada; 8 De dwa da dehs nye>s Aboriginal Health Centre, Hamilton, ON Canada

**Keywords:** Indigenous population health, Urban demographics, Health inequities, Barriers to health service access, Santé des populations autochtones, démographie urbaine, inégalités en matière de santé, obstacles à l’accès aux services de santé

## Abstract

**Objectives:**

Our Health Counts (OHC) methods are designed to address gaps in urban-based Indigenous health information. In partnership with local Indigenous health service providers, we have successfully implemented OHC in six Ontario cities. The aim of this study is to summarize findings regarding Indigenous population undercount, health inequities, and health service access barriers across study sites.

**Methods:**

We estimated Indigenous population size using OHC census participation survey responses and a multiplier approach. Health inequities between Indigenous populations and overall populations in each city were examined using respondent-driven sampling (RDS), adjusted OHC survey results, and existing public data. Measures included health status outcomes; determinants of health; barriers to health service access, including discrimination by health service providers; and unmet health needs.

**Results:**

Indigenous social networks were strong and extensive, and the urban populations demonstrate resilience and cultural continuity across multiple measures. Self-reported rates of census participation for Indigenous populations were markedly lower than those for the general population in each city, and OHC Indigenous population size estimates were consistently 2‒4 times higher than reported in the census. Indigenous to general population health inequities cut across measures of chronic disease, determinants of health, and unmet health needs. Indigenous populations experienced multiple barriers to health services access, including racial discrimination by health service providers.

**Conclusion:**

The Canadian census appears to markedly underestimate Indigenous population size in urban areas. Indigenous health inequities and service access barriers are striking and cross-cutting. Timely adaptation of health policies, services, and funding allocations in response to these findings is recommended.

## Résumé

**Objectifs:**

Les méthodes employées par Notre santé compte (NSC) sont conçues pour combler les lacunes dans les informations sur la santé des populations autochtones vivant en milieu urbain. En partenariat avec des prestataires de services de santé autochtone locaux, nous avons réussi à établir NSC dans six villes de l’Ontario. Notre étude résume les constatations ayant trait au sous-dénombrement des populations autochtones, aux iniquités en santé et aux obstacles à l’accès aux services de santé sur les lieux de l’étude.

**Méthode:**

Nous avons estimé la taille des populations autochtones à l’aide des réponses à l’enquête de NSC sur la participation au Recensement et d’une approche multiplicatrice. Au moyen d’un échantillonnage en fonction des répondants (EFR), des résultats ajustés de l’enquête de NSC et des données publiques existantes, nous avons examiné les iniquités en santé entre la population autochtone et la population globale de chaque ville. Les indicateurs employés ont été : les résultats en matière de santé; les déterminants de la santé; les obstacles à l’accès aux services de santé, dont la discrimination de la part des prestataires de services de santé; et les besoins de santé non comblés.

**Résultats:**

Les réseaux sociaux autochtones étaient vastes et solides, et les populations urbaines démontrent une résilience et une continuité culturelle à l’échelle de plusieurs indicateurs. Dans chaque ville, les taux autodéclarés de participation de la population autochtone au Recensement étaient nettement inférieurs à ceux de la population générale, et la taille de la population autochtone estimée par NSC était systématiquement de deux à quatre fois plus élevée que celle déclarée dans le Recensement. Les iniquités en santé entre les populations autochtones et la population générale transcendaient les indicateurs de maladie chronique, les déterminants de la santé et les besoins de santé non comblés. Les populations autochtones connaissaient de nombreux obstacles à l’accès aux services de santé, dont la discrimination raciale de la part des prestataires de services de santé.

**Conclusion:**

Le Recensement du Canada semble nettement sous-estimer la taille des populations autochtones vivant dans les agglomérations urbaines. Les iniquités en santé et les obstacles à l’accès aux services que connaissent les populations autochtones sont frappants et transversaux. Il est recommandé de réagir rapidement à ces constatations en adaptant les politiques et les services de santé et en affectant des fonds à la santé.

## Introduction

While many Indigenous peoples and communities are thriving despite colonial interventions, First Nations, Inuit, and Métis Peoples (FNIM) living in urban and related homelands of Canada continue to face a disproportionate burden of chronic health conditions and associated risk factors, health determinants, and barriers to safe, adequate healthcare, compared to the general population (Allan & Smylie, 2015; Wilk et al., 2018). These inequities are rooted in generations of racist colonial policies designed to displace and assimilate Indigenous peoples. This can be seen in forced relocations, mobility restrictions, and the theft of Indigenous children from their families (i.e., residential schools, the “Sixties Scoop”, the child welfare system) (Loppie Reading & Wien, 2009). In urban areas, a lack of comprehensive, population-responsive Indigenous-led health policy, coupled with jurisdictional ambiguities between provincial and federal governments responsible for providing health services, further drives these systemic disparities, resulting in gaps in care and service delivery (Browne, 2017; Smylie et al., 2011; Snyder et al., 2015).

While “urban areas are increasingly important in the life paths of Indigenous people”, and FNIM living in urban and related homelands demonstrate a strong sense of identity, relationship to community, and relationship to the land (Landry et al., 2019, p. 4781), urban-based health remains a key area of concern. FNIM peoples represent the fastest growing segment of the urban population in Canada. In Ontario, an estimated 70% of FNIM peoples live in metropolitan areas (Statistics Canada, 2016). As reported below, OHC studies show that the number of FNIM living in Ontario cities is higher than reported by the Census*.* While urban-based Indigenous populations are growing, there is no evidence that health disparities improve with urban residence, and Indigenous peoples are often excluded, misclassified, or under-represented in urban population health initiatives, data collection, and reporting (Smylie & Firestone, 2015). Census undercounts result from increased risk of non-participation among Indigenous and “hard-to-reach” populations. This is often due to systemic bias, a history of government distrust due to colonial policies, and sampling methods that miss mobile and homeless populations. Importantly, the inability to adequately identify Indigenous populations in healthcare databases leads to difficulty in identifying and addressing inequities in health status, health determinants, and healthcare access in urban areas. These information gaps create challenges for implementing appropriate, evidence-based health interventions and services (Smylie & Firestone, 2015).

Despite pervasive inequities in health determinants and outcomes, and ongoing Indigenous population growth in urban and related homelands, there is little comprehensive and inclusive data to inform and address urban-specific health and social needs (Rotondi et al., 2017). Incomplete health information limits data about population size, health, and well-being of FNIM peoples living in urban areas (Smylie et al., 2012). These data limitations are compounded by systemic barriers and colonial processes that prevent and exclude Indigenous people from governing, managing, and leading their own research and data processes (Well Living House, 2018). As a result, existing measures and health information systems are not representative of the urban-based Indigenous population, nor do they recognize the critical role of Indigenous leadership and community partnership. These systemic deficiencies in identifying FNIM peoples living in urban and related homelands in Canadian health information systems can impede population-based health assessments, which impact service planning and delivery (Smylie et al., 2018). In response to these data gaps and concerns, Our Health Counts (OHC) was initiated in 2008 to establish a community-governed, urban, Indigenous population health database system that would provide meaningful and culturally relevant data to communities (Beckett et al., 2018). OHC has since been successfully implemented in five additional urban areas: Ottawa, Toronto, London, Thunder Bay, and Kenora.

This study contributes to addressing critical gaps in Indigenous health data in urban and related homelands. The objectives of the paper are twofold: (1) to summarize and document findings regarding Indigenous population undercount, health inequities, measures of resilience, and health service access across six distinct OHC study sites: Hamilton, Ottawa, Toronto, London, Thunder Bay, and Kenora, and (2) to demonstrate the critical need for urban, Indigenous-led health information database systems.

## Methods

### Community research partnerships

Community partnerships are critical to generating valid Indigenous health data. OHC is a comprehensive health study built on Indigenous community research principles, ethics, and governance protocols. At each site, OHC was co-led by an Indigenous health service organization and the Well Living House Action Research Centre for Indigenous Infants, Children, and their Families’ Health and Well-being. Partners included the Ontario Federation of Indigenous Friendship Centres, De dwa da dehs nye > s Aboriginal Health Centre in Hamilton, Tungasuvvingat Inuit in Ottawa, Seventh Generation Midwives Toronto, Southwest Ontario Aboriginal Health Access Centre in London, Anishnawbe Mushkiki Aboriginal Health Access Centre in Thunder Bay, and Waasegiizhig Nanaandawe’iyewigamig Health Access Centre in Kenora. In all cases, the St. Michael’s Hospital Research Ethics Board granted ethics approval. At all sites, Indigenous community partners retain ownership and control of their data in accordance with the project’s academic-community partnership agreements.

### Respondent-driven sampling

OHC cohorts are recruited using respondent-driven sampling (RDS), an advanced chain referral sampling method developed to sample “hard-to-reach” populations for which existing sampling frames are inadequate (Ramirez-Valles et al., 2005). Briefly, RDS methods apply participant social network information and recruitment chain positioning to generate population-based estimates for indicators of interest (Johnston & Sabin, 2010). We have previously detailed its effectiveness in Indigenous contexts (Kitching et al., 2020; Rotondi et al., 2017; Smylie et al., 2011), which we attribute to its synergies with strong and persistent Indigenous social networks across OHC sites.

In each city, RDS was initiated by selecting a demographically diverse, well-networked sample of FNIM “seeds”. Each seed completed the survey and was given three referral coupons to distribute to friends, acquaintances, or family members. Subsequent participants (i.e., recruits) were given three recruitment coupons, and so on until target sample size was achieved.

**Fig. 1:**
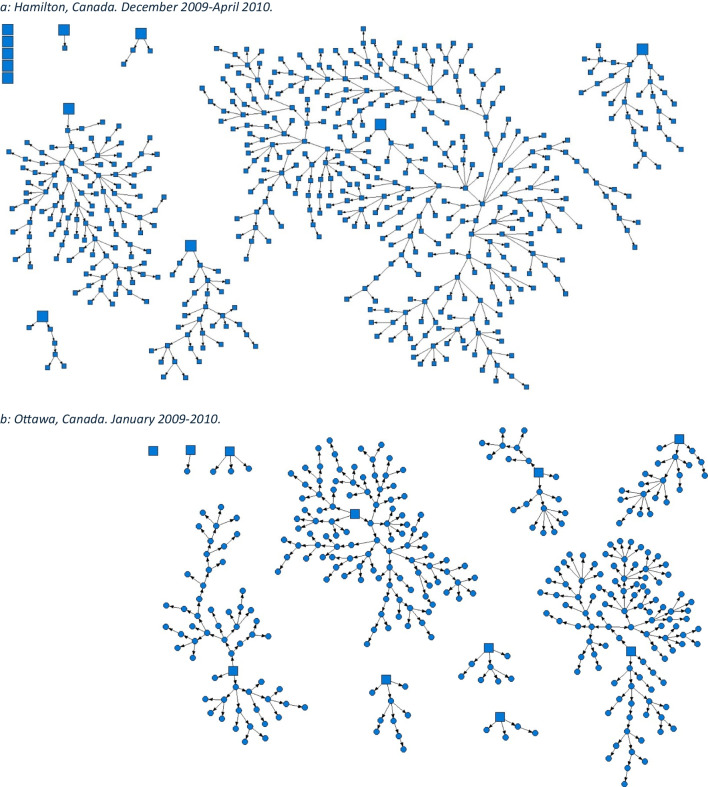

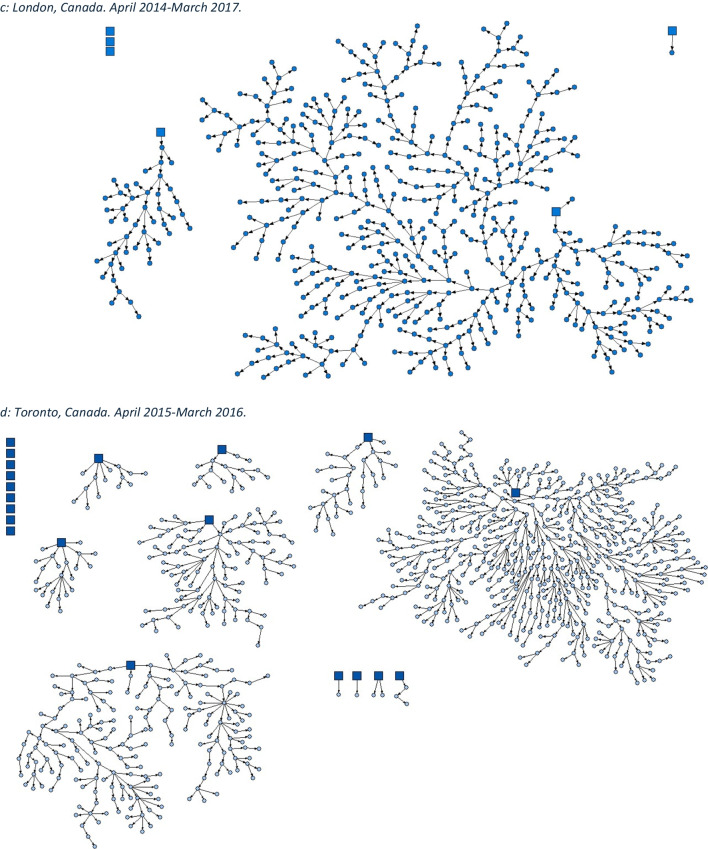

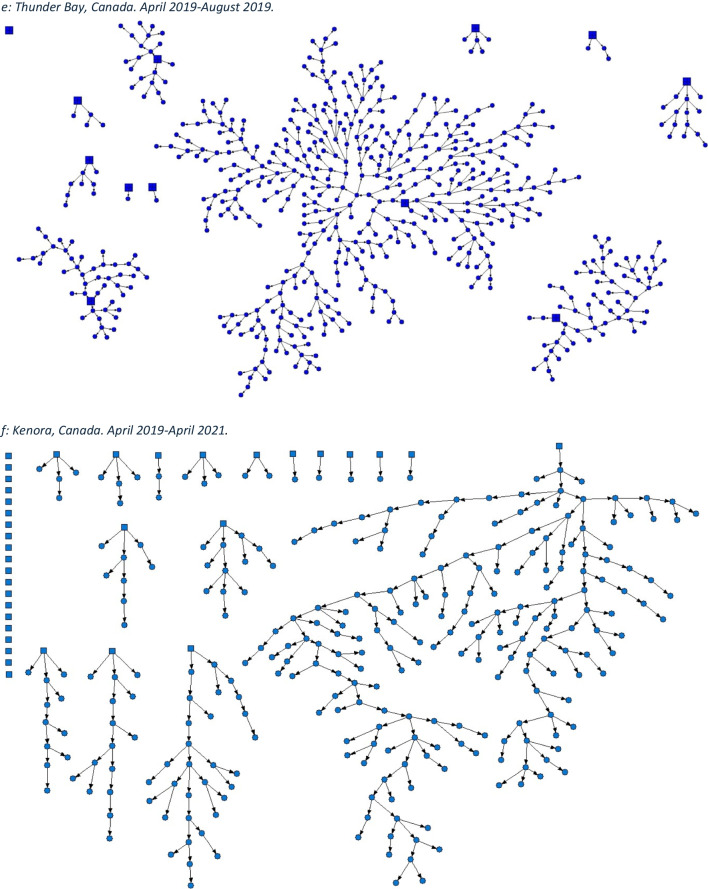
Strength of social networks and kin systems: RDS recruitment diagrams from OHC study sites. Seeds represented by squares, recruits represented by circles. **a** Hamilton, Canada. December 2009–April 2010. **b** Ottawa, Canada. January 2009–2010. **c** London, Canada. April 2014-March 2017. **d** Toronto, Canada. April 2015-March 2016. **e** Thunder Bay, Canada. April 2019–August 2019. **f** Kenora, Canada. April 2019–April 2021

We employed the dual incentive structure typical to RDS studies. Participants were provided $20 for survey completion and an additional $10 for each peer recruited (to a maximum of 5). At time of recruitment, all participants were asked for consent to have their data anonymously linked to administrative data at the Institute for Clinical Evaluative Sciences (ICES) using participants' Ontario Health Insurance Plan (OHIP) numbers. Comprehensive and locally tailored health assessment surveys, 60‒90 min in length, were conducted in a culturally safe manner by trained community interviewers. Site-specific RDS recruitment network diagrams (Fig. 1) were created using NetDraw software (Borgatti, 2002).

In Hamilton, inclusion criteria for adult participation included those ≥ 18 years of age (< 18 years if a parent), who self-identified as First Nations/Native/Indian, and resided in Hamilton. In Ottawa, inclusion criteria required that participants were ≥ 18 years of age (< 18 years if a parent), self-identified as Inuk, and resided in Ottawa. In the case of Toronto, London, Thunder Bay, and Kenora, the communities expanded inclusion criteria to capture adult participants ≥ 15 years of age (≥ 14 years if a parent), who identified as Indigenous (First Nations, Métis, and/or Inuit), and who resided in the city in question, and/or were the recipient of health, education, and/or social services in that city.

### Statistical analysis

Using the RDS Package in R Software (R Core Team, 2013) and SAS 9.4 software (SAS Institute Inc., 2013), RDS-II adjusted population-based estimates with 95% confidence intervals were produced by applying RDS-II weights to all descriptive analyses. RDS-II weights were calculated using the inverse of participant’s network sizes and applied to analyses to account for the unequal probability of recruitment due to participant’s varying network sizes (Salganik & Heckathorn, 2004).

A multiplier approach was used to calculate population size estimates. This approach draws on proportions of the sample who participated, did not participate, or did not know if they participated in the census, along with the census estimate of the FNIM population for the given survey year. It was assumed that only those who reported participation had indeed participated in the given census. Census estimates were then multiplied proportionally to correct for census non-participation, using the following formula: *N* = 1/*P* * *M* = *M/P*, 1 where *N* represents the estimated population size, *P* represents the RDS-adjusted proportion, and *M* represents the total number of those who reported completing the census (Johnston et al., 2013). Detailed methods for OHC population size estimates for individual OHC sites have been described elsewhere (Rotondi et al., 2017; Smylie et al., 2018).

Specifically, all OHC surveys with the exception of Hamilton included the question: “Did you complete the “year” Census Canada questionnaire?”. Using this indicator and publicly available Canadian census information, OHC population estimates were calculated in two ways: (1) a conservative approach which assumes those who respond “don’t know/don’t remember” completing the census, did complete it. This is unlikely, however, as community stakeholders report that respondents are more likely to answer “don’t know/don’t remember” rather than “no” due to fear of reprisal for not completing the legally required census; and (2), a non-conservative approach that assumes those who respond “don’t know/don’t remember” did not complete the census (Rotondi et al., 2017).

Population-based estimates of emergency room (ER) use and hospitalization rates among FNIM in each city were produced using direct linkage of each OHC cohort to ICES data holdings and comparator general city and provincial rates were produced using ICES data.

### Multi-group ethnic identity measure

A modified Multi-Group Ethnic Identity Measure (MEIM) score was used to measure connection to Indigenous identity. A MEIM score is produced using 12 self-rated questions that assess an individual’s connection to identity in the following areas: ethnicity, affirmation, belonging, and commitment (Phinney, 1992). For the OHC studies, “strong” was defined as having a mean MEIM score of at least 2.875 and “developing” as having a mean MEIM score of less than 2.874.

## Results

### Recruitment

Using RDS methods, OHC sites have successfully generated valid, population-representative cohorts of FNIM adults (Fig. 1). In Hamilton, 554 First Nations adults were recruited, and in Ottawa 341 Inuit adults were recruited. In Toronto, 908 Indigenous adults were recruited, in London the total sample size was 508, in Thunder Bay 601, and in Kenora 302. RDS leverages the strength of Indigenous social and kin networks which allows for the recruitment of a population-representative sample. OHC demonstrates the connectedness of community and the strength of FNIM networks.

### Population undercounts

Using conservative and non-conservative estimates, the number of Indigenous people residing in five of the OHC study sites was estimated to be at least 2‒4 times higher than the census would indicate (Table 1). For example, the 2011 census and National Household Survey reported that there were 15,650 Indigenous adults living in Toronto, Ontario. Using RDS, a conservative estimate shows that there are 55,000 Indigenous adults living in the city of Toronto, and a non-conservative estimate shows 74,000. This is 2‒4 times larger than the population size reported by Statistics Canada (Rotondi et al., 2017).

**Table 1:** Magnitude of Canadian census undercounts for FNIM adults (15 + years of age) across five OHC study sites

Study site	Statistics Canada adult population size estimates	OHC non-conservative adult population size estimates from RDS (95% CI)^a^	OHC conservative adult population size estimates^a,b^ from RDS (95% CI)	Population surveyed	Magnitude of adult population undercount
Toronto	15,650 (2011 Census and National Household Survey)	60,000 (47,000‒84,000)	45,000 (37,000‒59,000)	Self-identify as Indigenous	A factor of 2 to 4
Ottawa	605 (2006 Census)	3361 (2309‒4959)	1505 (1077‒2270)	Self-identify as Inuk	A factor > 4
London	8410 (2016 Census)	22,155 (16,646‒33,473)	17,108 (13,448‒23,508)	Self-identify as Indigenous	A factor of 3 to 4
Thunder Bay	9780 (2016 Census)	29,778 (23,080‒42,641)	13,800 (11,932‒17,604)	Self-identify as Indigenous	A factor of 2 to 4
Kenora	2235 (2016 Census)	9132 (5324‒32,121)	5985 (3954‒12,310)	Self-identify as Indigenous	A factor > 4

^a^OHC estimates are for the same year as the referent Statistics Canada population size estimates

^b^Don’t know/remember/unreliable assumed to have completed the census

### Health determinants

Striking disparities of health determinants were unmasked across survey sites (Tables 2, 3, 4, 5, 6, and 7). In Hamilton, 73.0% (95% CI, 66.5‒79.5) of the First Nations population earned a total annual personal income ≤ $20,000. In Ottawa, 70.3% (95% CI, 62.5‒78.0) of Inuit adults earned ≤ $20,000, and 39.8% (95% CI, 31.3‒48.3) were unemployed. Unemployment among the Inuit population was almost 9 times higher than that among the general Ottawa population (Smylie et al., 2012). Later OHC surveys used the Low-Income Cut-Off (LICO) to measure income. More than 8 in 10 Indigenous adults were living at or below the LICO in Toronto (86.9%; 95% CI, 83.1‒90.7), London (90.0%; 95% CI, 84.5‒94.9), and Thunder Bay (89.2%; 95% CI, 84.9‒93.5). In Kenora, 62.6% (95% CI, 53.3‒71.8) lived at or below the LICO. In Toronto, 63.0% (95% CI, 55.4‒68.6) of Indigenous adults were unemployed, as were 55.6% (95% CI, 47.5‒63.8) in London, 66.5% (95% CI, 60.5‒72.5) in Thunder Bay, and 65.1% (95% CI, 55.7‒74.6) in Kenora. In comparison, payroll employment increased by 2.2% for the general Ontario population, and 6.5% of the population were unemployed (Bourbeau & Fields, 2017).

**Table 2:** Measures of health determinants, health status, healthcare access, and resiliency among First Nations adults in Hamilton, Ontario (2010)

First Nations adults	RDS-II %	95% confidence interval
Health determinants		
Total personal income ≤ $20,000	73.0	66.5, 79.5
Completed high school/some college or more	45.0	36.7, 53.3
Precariously housed or homeless	11.9	6.3, 17.4
Had to give up important things (i.e., buying groceries) to meet shelter-related costs?	59.6	51.8, 67.4
Sometimes or often did not have enough to eat	20.8	14.2, 27.5
Health status		
Prevalence of the following health conditions:		
Diabetes	14.8	9.1, 20.5
High blood pressure	23.8	17.5, 30.1
Asthma	17.1	11.5, 22.7
Hepatitis C	8.8	4.5, 13.2
Learning disability	19.8	13.6, 25.9
Healthcare access		
Treated unfairly by a health professional because you are First Nations?	11.3	6.7, 15.8
Discrimination stopped, prevented, or delayed return to health services	62.7	45.7, 79.6
ER visit in previous 2 years	26.9	20.5, 33.3
Quality of ER care (fair/poor)	45.8	34.0, 57.7
Quality of hospital care (fair/poor)	39.8	27.8, 51.8
Measures of resilience and cultural continuity		
Social connections (community working together) a strength in community?	34.3	27.2, 41.4
Use traditional medicines	31.3	24.2, 38.3

**Table 3:** Measures of health determinants, health status, healthcare access, and resiliency among Inuit adults in Ottawa, Ontario (2010)

Inuit adults	RDS-II %	95% confidence interval
Health determinants		
Total personal income ≤ $20,000	70.3	62.5, 78.0
Unemployed	39.8	31.3, 48.3
Completed high school/some college or more	39.9	30.8, 48.9
Precariously housed or homeless	10.6	5.2, 16.02
Were there times when the food for you and your family did not last?	55.2	46.7, 63.7
Health status		
Prevalence of the following health conditions:		
Diabetes	4.9	1.6, 8.3
High blood pressure	27.8	19.7, 35.8
Asthma	15.2	9.3, 21.1
Hepatitis C	5.4	0.5, 10.3
Healthcare access		
Treated unfairly or kept waiting by a doctor, nurse, or dentist because of their identity	12.8	6.4, 19.2
ER visit in previous 2 years	60.4	52.6, 68.3
Quality of ER care (fair/poor)	22.9%	12.7, 33.1
Quality of hospital care (fair/poor)	40.7%	29.5, 52.0
Measures of resilience		
Social connections a strength in community?	59.7	51.4, 67.9

**Table 4:** Measures of health determinants, health status, healthcare access, and resiliency among FNIM adults in Toronto, Ontario (2016)

FNIM adults	RDS-II %	95% confidence interval
Health determinants		
Income at or below LICO	86.9	83.1, 90.7
Unemployed	63.0	57.4, 68.6
High school or more (age 25‒64)	56.6	50.4, 62.8
Precariously housed or homeless	34.9	29.8, 40.0
Had to give up important things (i.e., buying groceries) to meet shelter-related costs	59.2	53.0, 65.5
Sometimes or often did not have enough to eat	25.5	20.4, 30.6
Health status		
Prevalence of the following health conditions:		
Diabetes	14.9	10.8, 19.0
High blood pressure	23.8	18.7, 28.9
Asthma	24.3	19.4, 29.1
Hepatitis C	11.2	8.1, 14.3
Learning disability	21.6	17.3, 25.9
2 + chronic health conditions (age ≥ 20)	44.3	38.5, 50.2
Healthcare access		
Treated unfairly by a healthcare professional (Y)	28.1	23.0, 33.1
Discrimination stopped, prevented, or delayed return to health services?	71.4	61.6, 81.3
Unmet health needs	27.9	22.8, 33.0
Regular family doctor or nurse practitioner (Y)	62.9	57.5, 68.4
Accessed emergency care in past 12 months	46.4	40.7, 52.0
Quality of ER care (fair/poor)	41.6	33.3, 50.0
Quality of hospital care (fair/poor)	35.2	27.3, 43.2
Measures of resilience		
Ability to handle stress (E/VG/G)	69.5	64.1, 74.9
If answered Y to above (ability to handle stress):		
Strong sense of belonging to community	80.3	75.1, 85.5
Cultural continuity		
Speak Indigenous language(s)	41.2	35.7, 46.7
Participate in ceremony	65.0	59.3, 70.7
Use traditional medicines	49.6	43.9, 55.2
Eat traditional foods	50.5	44.8, 56.2
MEIM score (high/low)	High: 71.1; Low: 28.9	66.0, 76.1; 23.9, 34.0

**Table 5:** Measures of health determinants, health status, healthcare access, and resiliency among FNIM adults in London, Ontario (2017)

FNIM adults	RDS-II %	95% confidence interval
Health determinants		
Income at or below LICO	90.0	84.5, 94.9
Unemployed	55.6	47.5, 63.8
High school or more	53.8	44.4, 63.1
Precariously housed or homeless	21.9	15.5, 28.3
Had to give up important things (i.e., buying groceries) to meet shelter-related costs?	53.7	45.3, 62.2
Sometimes or often did not have enough to eat	21.2	15.2, 27.3
Health status		
Prevalence of the following health conditions:		
Diabetes	14.8	9.5, 20.1
High blood pressure	17.3	13.1, 21.5
Asthma	19.9	13.5, 26.4
Hepatitis C	4.5	2.5, 6.5
Learning disability	15.1	9.1, 21.1
Multi-morbidity (age ≥ 20)	30.2	23.4, 37.0
Healthcare access		
Treated unfairly by a healthcare professional (Y)	26.1	19.6, 32.5
Did discrimination stop, prevent, or delay return to health services?	66.6	54.7, 78.6
Unmet health needs	26.3	19.9, 32.7
Regular family doctor or nurse practitioner (Y)	64.8	57.4, 72.2
Accessed emergency care in past 12 months	32.5	25.7, 39.4
Quality of ER care (fair/poor)	30.7	20.0, 41.3
Quality of hospital care (fair/poor)	20.6	12.5, 28.6
Measures of resilience		
Ability to handle stress (E/VG/G)	67.6	60.7, 74.4
If answered Y to above (ability to handle stress):		
Strong sense of belonging to community	78.9	70.0, 87.8
Cultural continuity		
Speak Indigenous language(s)	49.0	41.1, 56.9
Participate in ceremony	66.1	58.4, 73.7
Use traditional medicines	62.3	54.7, 69.9
Eat traditional foods	65.5	58.3, 72.7
MEIM score (high/low)	High: 68.7; Low: 31.3	61.2, 76.3; 23.7, 38.9

**Table 6:** Measures of health determinants, health status, healthcare access, and resiliency among FNIM adults in Thunder Bay, Ontario (2019)

FNIM adults	RDS-II %	95% confidence interval
Health determinants		
Income at or below LICO	89.2	84.9, 93.5
Unemployed	66.5	60.5, 72.5
High school or more	32.5	25.9, 39.1
Precariously housed or homeless	24.7	18.9, 30.5
Had to give up important things (i.e., buying groceries) to meet shelter-related costs?	59.4	52.1, 66.8
Sometimes or often did not have enough to eat	28.1	21.9, 34.3
Health status		
Prevalence of the following health conditions:		
Diabetes^a^	14.9	10.3, 19.5
High blood pressure	21.2	15.7, 26.8
Asthma	12.0	7.9, 16.1
Hepatitis C	14.4	10.2, 18.5
Learning disability	20.8	15.9, 25.6
Multi-morbidity (age ≥ 20)	44.1	37.4, 50.7
Healthcare access		
Treated unfairly by a healthcare professional (Y)	39.3	33.0, 45.6
Did discrimination stop, prevent, or delay return to health services?	65.5	55.8, 75.3
Unmet health needs	22.7	17.2, 28.3
Regular family doctor or nurse practitioner (Y)	50.5	44.0, 56.3
Accessed emergency care in past 12 months	46.1	39.6, 52.6
Quality of ER care (fair/poor)	48.7	39.4, 58.1
Quality of hospital care (fair/poor)	40.8	32.1, 49.5
Measures of resilience		
Ability to handle stress (E/VG/G)	71.8	65.9, 77.6
If answered Y to above (ability to handle stress):		
Strong sense of belonging to community	79.4	72.7, 86.1
Cultural continuity		
Speak Indigenous language(s)	41.0	34.6, 47.4
Participate in ceremony	53.1	46.6, 59.7
Use traditional medicines	39.8	33.5, 46.1
Eat traditional foods	64.8	58.4, 71.3
MEIM score (high/low)	High: 81.8; Low: 18.2	74.5, 89.1; 11.0, 25.5

^a^Diabetes variable includes pre-diabetes, glucose intolerance, or diabetes

**Table 7:** Measures of health determinants, health status, healthcare access, and resiliency among FNIM adults in Kenora, Ontario (2021)

FNIM adults	RDS-II %	95% confidence interval
Health determinants		
Income at or below LICO	62.6	53.3, 71.8
Unemployed	65.1	55.7, 74.6
High school or more	35.8	25.2, 46.4
Precariously housed or homeless	30.5	21.6, 39.4
Had to give up important things (i.e., buying groceries) to meet shelter-related costs?	39.8	27.8, 51.9
Sometimes or often did not have enough to eat	14.9	8.0, 21.8
Health status		
Prevalence of the following health conditions:		
Diabetes^a^	12.8	5.9, 19.6
High blood pressure	27.2	19.9, 34.4
Asthma	24.5	17.6, 31.4
Hepatitis C	7.1	3.1, 11.0
Learning disability	12.8	6.6, 18.9
Multi-morbidity (age ≥ 20)	33.8	25.1, 42.5
Healthcare access		
Treated unfairly by a healthcare professional (Y)	38.7	29.8, 47.6
Did discrimination stop, prevent, or delay return to health services?	37.1	24.1, 50.1
Unmet health needs	11.1	6.2, 15.9
Regular family doctor or nurse practitioner (Y)	63.0	53.9, 72.0
Accessed emergency care in past 12 months	42.6	32.9, 52.2
Quality of ER care (fair/poor)	36.4	24.8, 48.0
Quality of hospital care (fair/poor)	15.0	5.1, 24.9
Measures of resilience		
Ability to handle stress (E/VG/G)	57.3	48.0, 66.5
If answered Y to above (ability to handle stress):		
Strong sense of belonging to community	88.2	77.8, 98.7
Cultural continuity		
Speak Indigenous language(s)	40.1	30.9, 49.2
Participate in ceremony	75.6	66.6, 84.6
Use traditional medicines	51.0	41.3, 60.8
Eat traditional foods	75.8	68.6, 83.0
MEIM score (high/low)	High: 54.2; Low: 45.8	44.0, 64.4; 35.5, 56.0

^a^Diabetes variable includes pre-diabetes, glucose intolerance, or diabetes

Income and employment disparities affect access to housing, food security, and educational opportunities. In terms of education, 45.0% (95% CI, 36.7‒53.3) of First Nations adults in Hamilton and 39.9% (95% CI, 30.8‒48.9) of Inuit adults in Ottawa had completed high school or more. In Toronto, 56.6% (95% CI, 50.4‒62.8) of Indigenous adults had completed high school or more, as had 53.8% (95% CI, 44.4‒63.1) in London, 32.5% (95% CI, 22.3‒42.8) in Thunder Bay, and 35.8% (95% CI, 25.2‒46.4) in Kenora. In comparison, 87.9% of the overall Ontario adult population has completed high school or more (Statistics Canada, 2016).

Consistently, more than half of FNIM adults living in the included Ontario cities reported that financial hardship had affected their overall health and well-being. In Hamilton, 11.9% (95% CI, 6.3‒17.4) of First Nations adults were precariously housed or homeless (defined as homeless, in transition, or living in any other type of dwelling not listed). Food security was also an issue. Approximately two thirds (59.6%; 95% CI, 51.8‒67.4) of the population had to give up important things (i.e., buying groceries) to meet shelter-related costs and 20.8% (95% CI, 14.2‒27.5) sometimes or often did not have enough to eat. In Toronto, 1 in 3 (34.9%; 95% CI, 29.8‒40.0) Indigenous adults were homeless or precariously housed, 59.2% (95% CI, 53.0‒65.5) had to give up important things to meet shelter-related costs, and 25.5% (95% CI, 20.4‒30.6) sometimes or often did not have enough to eat. In London, the estimated proportions were 21.9% (95% CI, 15.5‒28.3), 53.7% (95% CI, 45.3‒62.2), and 21.2% (95% CI, 15.2‒27.3), respectively. In Thunder Bay, the estimated proportions were 24.7% (95% CI, 18.9‒30.5), 59.4% (95% CI, 52.1‒66.8), and 28.1% (95% CI, 21.9‒34.3), respectively; and in Kenora, the estimated proportions were 30.5% (95% CI, 21.6‒39.4), 39.8% (95% CI, 27.8‒51.9), and 14.9% (95% CI, 8.0‒21.8), respectively.

### Health status

A higher proportion of FNIM adults experienced multimorbidity (≥ 2 chronic health conditions) compared to the general population. In Toronto, 44.3% (95% CI, 38.5‒50.2) experienced multimorbidity. In London, Thunder Bay, and Kenora, 30.2% (95% CI, 23.4‒37.0), 44.1% (95% CI, 37.4‒50.7), and 33.8% (95% CI, 25.1‒42.5), respectively, experienced multimorbidity. In comparison, 12.9% (95% CI, 12.6‒13.2) of the general Canadian population, 20 + years of age, experience multimorbidity (Roberts et al., 2015).

The most reported chronic health conditions diagnosed by a healthcare provider were consistent across all OHC study sites: asthma, hypertension, and diabetes. These are also commonly reported chronic health conditions for the general population; the difference lies in the prevalence of these conditions, and in access to timely adequate care. Public data from the Canadian Community Health Survey are used to contrast the prevalence of specific chronic health conditions below.

Self-reported diabetes prevalence was notably high across all study sites, apart from Ottawa, where 4.9% (95% CI, 1.6‒8.3) of Inuit adults had been diagnosed with diabetes. For example, 15.6% (95% CI, 11.2‒21.1) of First Nations adults in Hamilton had been diagnosed with diabetes. This was approximately three times the rate among the general population (CCHS, 2007), despite a younger age demographic among the First Nations population. In Thunder Bay, diabetes prevalence was closer to that of the general population (CCHS, 2015).

Rates of hypertension are also elevated for FNIM populations living in urban and related homelands. In some cases, this difference was notable: for example, in Ottawa, 27.8% (95% CI, 19.7‒35.8) of urban Inuit reported having high blood pressure compared to 13.8% of Ottawa adults (CCHS, 2007).

Asthma was another commonly reported chronic health condition. The most notable comparison is seen in Toronto, where the prevalence of asthma in Indigenous adults (24.3%; 95% CI, 19.4‒29.1) is more than triple that of the general urban population (7.7%). In Thunder Bay, rates were closer to that of the general population (CCHS, 2015).

Health inequalities are also seen in hepatitis C rates. In Hamilton, the prevalence of hepatitis C was 8.8% (95% CI, 4.5‒13.2), over 10 times the provincial rate (0.8%). In Toronto, 11.2% (95% CI, 8.1‒14.3), in London, 4.5% (95% CI, 2.5‒6.5), in Thunder Bay, 14.4% (95% CI, 10.2‒18.5), and in Kenora, 7.1% (95% CI, 3.1‒11.0) of Indigenous adults had been diagnosed with hepatitis C. In comparison, approximately 1% of Canadian adults were diagnosed with this chronic illness (Well Living House, 2018).

With respect to learning disabilities, 21.6% (95% CI, 17.3‒25.9) of Indigenous adults in Toronto had been diagnosed with a learning disability. In London, 15.1% (95% CI, 9.1‒21.1), in Thunder Bay, 20.8% (95% CI, 15.9‒20.6), and in Kenora, 12.8% (95% CI, 6.6‒18.9) had been diagnosed compared to 4% of Canadian adults (CSD, 2017).

### Measures of resilience and cultural continuity

Notwithstanding multi-generational challenges to health and wellness, FNIM adults demonstrate strength of community social networks, identity, cultural continuity, and resilience across OHC study sites (Tables 2, 3, 4, 5, 6, and 7). The strength of social networks was demonstrated by the success of RDS methods, which highlight expansive and cross-cutting Indigenous social networks in each city (Smylie et al., 2012). Approximately two thirds of Indigenous adults felt their ability to handle stress was excellent, very good, or good (Toronto, 69.5% (95% CI, 64.1‒74.9); London, 67.6% (95% CI, 60.7‒74.4); Thunder Bay, 71.8% (95% CI, 65.9‒77.6); Kenora, 57.3% (95% CI, 48.0‒66.5)). Approximately 8 in 10 of those who rated their ability to handle stress as excellent, very good, or good also expressed a strong sense of belonging to Indigenous community (Toronto, 80.3% (95% CI, 75.1‒85.5); London, 78.9% (95% CI, 70.0‒87.8); Thunder Bay, 77.3% (95% CI, 71.7‒83.0); Kenora, 88.2% (95% CI, 77.8‒98.7)). With respect to cultural continuity, more than 4 in 10 FNIM adults spoke an Indigenous language across all study sites. In Toronto, London, and Kenora, more than two thirds of FNIM adults participated in ceremony, and in Thunder Bay, over 50% did. Overall, most also used traditional medicines and ate traditional foods (Table 8). Further, cross-site findings indicate a total MEIM identity score that reflects a strong sense of identity among FNIM adults across urban and related homelands (Tables 4, 5, 6, and 7).

**Table 8:** Measures of cultural continuity among FNIM adults (15 + years of age) in four Ontario cities, RDS-II % (95% confidence interval)

Study site	Speak Indigenous language(s)	Participate in ceremony	Use traditional medicines	Eat traditional foods
Toronto	41.2% (35.7, 46.7)	65.0% (59.3, 70.7)	49.6% (43.9, 55.2)	50.5% (44.8, 56.2)
London	49.0% (41.1, 56.9)	66.1% (58.4, 73.7)	62.3% (54.7, 69.9)	65.5% (58.3, 72.7)
Thunder Bay	41.0% (34.6, 47.4)	53.1% (46.6, 59.7)	39.8% (33.5, 46.1)	64.8% (58.4, 71.3)
Kenora	40.1% (30.9, 49.2)	75.6% (66.6, 84.6)	51.0% (41.3, 60.8)	75.8% (68.6, 83.0)

### Health service access

The most common cross-site barriers to accessing health services included long waiting lists, lack of accessible/affordable transportation, provider unavailable, and lack of trust in healthcare provider. Discrimination from healthcare providers was a prevalent barrier to care. In Hamilton, adults were asked if they had been treated unfairly by a health professional because they were First Nations: 11.3% (95% CI, 6.7‒15.8) indicated that they had, and 62.7% (95% CI, 45.7‒79.6) reported that this had stopped or delayed them from returning to a health service. In Ottawa, 12.8% (95% CI, 6.4‒19.2) of Inuit reported that they were treated unfairly or kept waiting by a doctor, nurse, or dentist because of their identity. Approximately 1 in 3 FNIM adults reported that they had been treated unfairly by a healthcare professional because of their Indigenous identity: Toronto (28.1%; 95% CI, 23.0‒33.1), Thunder Bay (39.3%; 95% CI, 33‒45.6), London (26.1%; 95% CI, 19.6‒32.5), and Kenora (38.7%; 95% CI, 29.8‒47.6). In most cases, this led to delays in care: FNIM adults in Toronto (71.4%; 95% CI, 61.6‒81.3), London (66.6%; 95% CI, 54.7‒78.6), Thunder Bay (65.5%; 95% CI, 55.8‒75.3), and Kenora (37.1%; 95% CI, 24.1‒50.1) indicated that discrimination had stopped, prevented, or delayed their return to health services.

Further, more than 1 in 5 FNIM adults in Toronto (27.9%; 95% CI, 22.8‒33.0), London (26.3%; 95% CI, 19.9‒32.7), and Thunder Bay (22.7%; 95% CI, 17.2‒28.3) reported unmet health needs. In Kenora, 11.1% (95% CI, 6.2‒15.9) reported unmet health needs. In the case of Toronto, London, and Thunder Bay, this is approximately four times higher than that of the general population, where unmet health needs were reported among 6.2% of adults in Toronto, 5.5% in London, and 7.1% in Thunder Bay (CCHS, 2007, 2015).

When asked if they had a regular healthcare provider (i.e., family doctor or nurse practitioner), approximately two thirds of FNIM adults in Toronto (62.9%; 95% CI, 57.5‒68.4), London (64.8%; 95% CI, 57.4‒72.2), and Kenora (63.0%; 95% CI, 53.9‒72.0) had a regular provider, and 50.5% (95% CI, 44‒56.3) of FNIM adults in Thunder Bay did. In comparison, approximately 90% of the general population had a regular healthcare provider.

Rates of self-reported emergency room (ER) admission, for acute and non-acute conditions, were elevated across all OHC study sites. In Hamilton, 26.9% (95% CI, 20.5‒33.3) of First Nations adults reported visiting the ER at least once in the previous 2 years. Of those who had accessed emergency care, 45.8% (95% CI, 34.0‒57.7) felt the care they received was fair or poor. In Ottawa, 60.4% (95% CI, 52.6‒68.3) of Inuit adults reported visiting the ER in the previous 2 years, a rate 10 times that of the general Ottawa population (Smylie et al., 2012). Of those who had received emergency care, 22.9% (95% CI, 12.7‒33.1) of Inuit adults reported that the quality of care was fair or poor. In Toronto, 46.4% (95% CI, 40.7‒52.0) of FNIM adults reported accessing emergency care in the 12 months prior to the survey; in London, 32.5% (95% CI, 25.7‒39.4); in Thunder Bay, 46.1% (95% CI, 39.6‒52.6); and in Kenora, 42.6% (95% CI, 32.9‒52.2). In all cases, rates of ER access were approximately double the rate of the general Ontario population, 19% of whom accessed emergency care. Of the FNIM adults who accessed emergency care in Toronto, 41.6% (95% CI, 33.3‒50.0) rated the quality of care as fair/poor. Similarly, 30.7% (95% CI, 20.0‒41.3) of FNIM adults in London, 48.7% (95% CI, 39.4‒58.1) in Thunder Bay, and 36.4% in Kenora reported fair/poor quality healthcare.

The ICES data linkages further substantiate these striking disparities. According to the linked data, 68.5% of First Nations adults in Hamilton had accessed the ER at least one time in the previous 2 years, compared to 33.7% of the general Hamilton population (Smylie et al., 2011), and 55.4% of the Inuit population in Ottawa had visited an ER at least one time in the past 2 years, as compared to 31.1% of the general Ottawa population (Smylie et al., 2012). Between 2016 and 2018, 64.2% of FNIM adults in Toronto accessed the ER at least once, compared to 30.2% of the general Toronto population. Based on the data from the ICES linkage, rates of ER use among FNIM across all sites were approximately double the rates of the general city populations (Table 9).

**Table 9:** Self-reported and ICES data linkage emergency room (ER) use rates among FNIM in three Ontario cities compared to general city populations

Study site	Self-reported ER use among FNIM RDS-adjusted % (95% CI)	ICES linkage ER use rates among FNIM RDS-adjusted %	City comparator population
Hamilton	26.9 (20.5, 33.3)	68.5%	33.7%
Ottawa	60.4 (52.6, 68.3)	55.4%	31.1%
Toronto	46.4 (40.7, 52.0)	64.2%	30.2%

High rates of hospitalizations among FNIM are also present. Self-reported rates of hospitalizations across sites include Hamilton (42.3%; 95% CI, 34.7‒49.9), Ottawa (57.3%; 95% CI, 48.9‒65.7), Toronto (44.6%; 95% CI, 38.9‒50.3), London (39.6%; 95% CI, 31.9‒47.2), Thunder Bay (48.0%; 95% CI, 41.6‒54.4), and Kenora (44.1%; 95% CI, 34.6‒53.5). FNIM adults who had spent one or more nights in a hospital were asked to rate the quality of hospital care they received. In Hamilton, 39.8% (95% CI, 27.5‒51.8) rated their quality of care as fair/poor; in Ottawa, it was 40.7% (95% CI, 29.5‒52.0); in Toronto, 35.2% (95% CI, 27.3‒43.2); in London, 20.6% (95% CI, 12.5‒28.6); in Thunder Bay, 40.8% (95% CI, 32.1‒49.5); and in Kenora, 15.0% (95% CI, 5.1‒24.9).

Results from the ICES data linkage also validated survey response findings regarding hospitalization rates. In Hamilton, 26.0% of First Nations adults were hospitalized in the past 5 years, compared to 17.1% of the general Hamilton population (Smylie et al., 2011). In Ottawa, 20.5% of Inuit adults were hospitalized, compared to 14% of the general Ottawa population (Smylie et al., 2012). In Toronto, 45.0% of FNIM were hospitalized in comparison to 31.7% of the general city population. Across all sites, rates of hospitalizations were higher among FNIM in comparison to the general population (Table 10).

**Table 10:** Self-reported and ICES data linkage hospitalization rates among FNIM in three Ontario cities compared to general city populations

Study site	Self-reported hospitalizations among FNIM RDS-adjusted % (95% CI)	ICES linkage hospitalization rates among FNIM RDS-adjusted %	City comparator population
Hamilton	42.3% (34.7, 49.9)	26.6%	17.1%
Ottawa	57.3% (48.9, 65.7)	20.5%	14.0%
Toronto	44.6% (38.9, 50.3)	45.0%	31.7%

## Discussion

Our paper summarizes the results of the OHC study regarding Indigenous population undercounts; inequities in health determinants, health status, and service access; and measures of resilience and cultural continuity across six Ontario cities: Hamilton, Ottawa, Toronto, London, Thunder Bay, and Kenora. The study reveals cross-cutting and striking socio-economic and health disparities, and substantive barriers in access to safe, adequate healthcare. Additionally, this study highlights strong social networks, cultural continuity, and resilience among these populations. While Canada’s attempts at assimilation have not been successful, the implementation of colonial policies has negatively influenced structural determinants of health. The Truth and Reconciliation Commission (TRC) identifies the importance of acknowledging the direct linkage of Indigenous health disparities to colonial policies (TRC, 2015).

Applying RDS to the health needs assessment of FNIM peoples living in urban and related homelands has unmasked significant population undercounts in urban Ontario. OHC population estimates show that the Indigenous population at each study site is 2‒4 times greater than estimated by the Canadian census. These population count discrepancies point towards an urgent need to work in partnership with FNIM communities and service organizations to accurately enumerate urban-based Indigenous populations. There is a pressing need to ensure that policy and service responses, including but not limited to funding, are adjusted to updated population estimates that account for substantive census under-participation among FNIM peoples living in Canadian cities.

Across all six study sites, we see marked disparities in core determinants of health, including access to income, employment, education, housing stability, and food security. When compared to the general population, these disparities are particularly striking. For example, more than six times as many FNIM adults in urban and related homelands live at or below the LICO compared to the general Ontario population.

Findings show a disproportionate burden of chronic illness, which includes elevated rates of diabetes, hypertension, and asthma, as well as hepatitis C and learning disabilities across sites. We note a lower prevalence of diabetes among Inuit in Ottawa, which is likely reflective of the lower prevalence of diabetes among Inuit as compared with First Nations. This may in turn be rooted in a relatively shorter period of exposure to the diet and lifestyle changes imposed by European colonization. Relatively lower prevalences of all three chronic diseases for FNIM in Thunder Bay may be partially explained by the fact that FNIM in Thunder Bay also had the lowest rate of access to a regular primary care provider relative to other OHC sites, and our chronic disease question enquires about diagnosis by a healthcare provider.

Despite the youthfulness of the population, a notably high proportion of FNIM adults also experience multimorbidity (approximately 40% across Toronto, London, Thunder Bay, and Kenora), in particular when compared to the general population (which is relatively older), 12.9% of whom experience multimorbidity according to Roberts et al. (2015). This is of concern, given that multimorbidity is often associated with complex health outcomes, clinical management, and healthcare needs (Valderas et al., 2009), and that FNIM peoples living in cities experience barriers to safe, adequate care.

Despite living in urban areas with extensive health services and facilities, OHC findings show that FNIM adults experience cross-cutting healthcare barriers, including discrimination from healthcare providers. Approximately one third of FNIM adults reported discrimination in a healthcare setting, and of those who did, approximately two thirds felt it delayed or prevented their return to health services. While the proportion of FNIM adults in Hamilton, Ottawa, and Kenora who reported discrimination and care delays is lower, this should be interpreted with caution, as under-reporting experiences of racism is common (Krieger, 2014) and discrimination in healthcare settings results in negative health outcomes (Reader & Gillespie, 2013). Analysis of the Toronto OHC data demonstrates that discrimination by a healthcare provider is associated with significantly higher odds of unmet health needs (Kitching et al., 2020). Those who experience discrimination are less likely to feel safe seeking medical help (Smylie et al., 2012). As such, experiences of discrimination reduce access to care, and negatively impact patient health (Kitching et al., 2020; Reader & Gillespie, 2013).

More than 1 in 5 FNIM adults also reported unmet health needs, and rates of ER admission were elevated in each city. Disproportionately high rates of ER use are indicative of poor access to primary healthcare services. Racism and compounding barriers to accessing non-emergent healthcare may be linked to higher rates of ER usage in urban-based Indigenous populations (Smylie et al., 2018). Of concern, poor health status is linked to healthcare systems which lack culturally safe care.

These striking gaps are unsurprising given ongoing inadequacies in the scope of policy and funding support for Indigenous-specific primary care, relative to the population size. For example, in Toronto, there is only one provincially supported Indigenous-specific community health centre for a FNIM population currently estimated to be 88,000 (Smylie et al., 2022a). This clearly indicates an opportunity to apply needs and population-based primary care planning approaches to bridge these gaps and advance upstream, culturally safe health service models.

Notwithstanding these significant inequities, FNIM peoples living in participant cities demonstrate strong social networks, cultural identity, cultural continuity, and resilience. A notable proportion of FNIM peoples living in urban and related homelands speak an Indigenous language, actively participate in ceremony, use traditional medicines, and eat traditional foods. Most adults felt their ability to handle stress was excellent, very good, or good. These findings are consistent with literature challenging the narrative that cultural assimilation is an inevitable consequence of urban residency (e.g., Neale, 2017).

### Limitations of the study

There are some limitations to consider. RDS estimates are unbiased to the degree that the assumptions of the RDS estimator are met. That said, the unique sampling design of the OHC study sites has proven a valid and asymptotically unbiased representation of FNIM peoples living in urban and related homelands (Beckett et al., 2018; Smylie et al., 2022b). Extensive post-sampling evaluation including convergence and bottleneck plots that examine the distribution of key demographic indicators have shown stability of our estimates, which suggests cohorts are representative of the target populations (Smylie et al., 2022b). Survey findings may also be subject to self-reporting bias. While self-reporting surveys allow for a wider range of responses compared to other data collection instruments, they can introduce social desirability or recall bias. Additionally, the design effect of RDS methods translates into reduced precision for a given sample size, compared to random sampling methods, which unfortunately are not an option for these populations. This design effect limits our ability to produce cross-tabulations and has resulted in less precision than desirable for some prevalence estimates, particularly in OHC sites with smaller sample sizes. Notwithstanding, this study has several strengths, one of which is ongoing community partnership in research design, delivery, governance, and dissemination. Additionally, the replication and/or similarity of findings across study sites makes it unlikely that findings are spurious and brings important attention to key health and health service challenges and opportunities for FNIM peoples living in urban and related homelands. It also convincingly demonstrates Canadian census undercounting of FNIM populations in cities.

## Conclusion

Our Health Counts has established the efficacy of culturally safe, community-led and owned health databases for FNIM living in cities. The OHC model demonstrates scalability across diverse urban contexts, suggesting it could provide a model for data gathering and governance in other urban-based Indigenous communities. In summary, these findings point to the need to adapt and decolonize health policies, allocate appropriate funding for Indigenous service delivery, and implement cultural safety training for healthcare providers to understand the internalized biases that lead to discriminatory practices in care.

## Contributions to knowledge

What does this study add to existing knowledge?

- Our Health Counts (OHC) contributes to addressing critical gaps in First Nations, Inuit, and Métis (FNIM) health databases in urban and related homelands. This is the first study to examine cross-cutting findings across six distinct OHC study sites: Hamilton, Ottawa, Toronto, London, Thunder Bay, and Kenora.
- Findings demonstrate that the Canadian census underestimates FNIM population size in urban areas. OHC Indigenous population size estimates were consistently 2‒4 times higher than reported in the census.
- This study unmasks striking cross-site health inequities and barriers to health service access, including discrimination by healthcare providers.

What are the key implications for public health interventions, practice, or policy?

- Timely adaptation of health policies, services, and funding allocations in response to the study findings is recommended.
- OHC has established the efficacy of culturally safe, community-led and owned health databases for FNIM living in cities. The OHC model demonstrates scalability across urban contexts: it can provide a model for data gathering and governance in other Indigenous communities.
- Findings point to the need to adapt and decolonize health policies, allocate appropriate funding for Indigenous service delivery, and mandate cultural safety training for healthcare providers to understand the internalized biases that lead to discriminatory practices in care.

## Data Availability

The datasets used for analyses during the current study are not publicly available due to data sharing agreements between the community research partners and investigators.

## Code availability

N/A.
