# Correction: Unmasking population undercounts, health inequities, and health service access barriers across Indigenous populations in urban Ontario

**DOI:** 10.17269/s41997-025-01040-6

**Published:** 2025-05-29

**Authors:** Marcie Snyder, Stephanie McConkey, Raman Brar, Judy Anilniliak, Cheryllee Bourgeois, Brian Dokis, Michael Hardy, Serena Joseph, Amanda Kilabuk, Jo-Ann Mattina, Constance McKnight, Janet Smylie

**Affiliations:** 1 Well Living House, St. Michael’S Hospital, Unity Health Toronto, Toronto, ON Canada; 2 Tungasuvvingat Inuit, Ottawa, ON Canada; 3 Seventh Generation Midwives Toronto, Toronto, ON Canada; 4 Southwest Ontario Aboriginal Health Access Centre, London, ON Canada; 5 Anishnawbe Mushkiki Aboriginal Health Access Centre, Thunder Bay, ON Canada; 6 Waasegiizhig Nanaandawe’iyewigamig Aboriginal Health Access Centre, Kenora, ON Canada; 7 De dwa da dehs nye>s Aboriginal Health Centre, Hamilton, ON Canada; 8 Dalla Lana School of Public Health and Department of Family & Community Medicine, University of Toronto, Toronto, ON Canada

**Correction: Canadian Journal of Public Health (2024) 115:209–226**

10.17269/s41997-024-00957-8

This article was updated to correct mislabelling in the caption and on the graphics for Figure 1. In the figure caption, “c. Toronto, Canada. April 2015–March 2016.” was changed to “c. London, Canada. April 2014–March 2017.”, and “d. London, Canada. April 2014–March 2017.” was changed to “d. Toronto, Canada. April 2015–March 2016.” On the graphics themselves, the caption “*c) Toronto, Canada. April 2015–March 2016.*” was changed to “*c) London, Canada. April 2014*-*–March 2017.*”, and the caption “d) *London, Canada. April 2014–March 2017.*” was changed to “*d) Toronto, Canada. April 2015–March 2016*”.

Both versions of the figures and legends appear on the following pages for clarity.

**[Original] Fig. 1:**
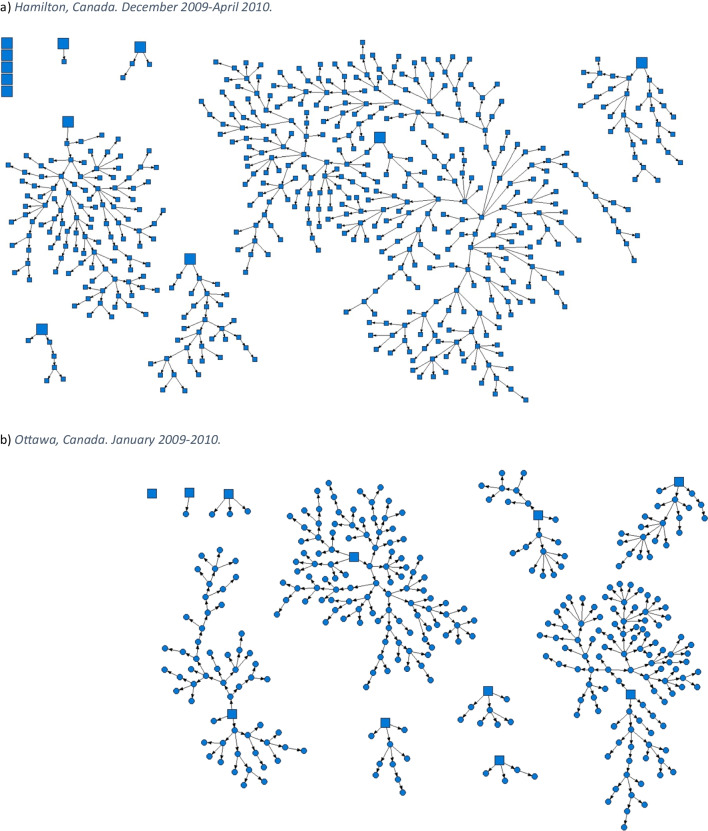

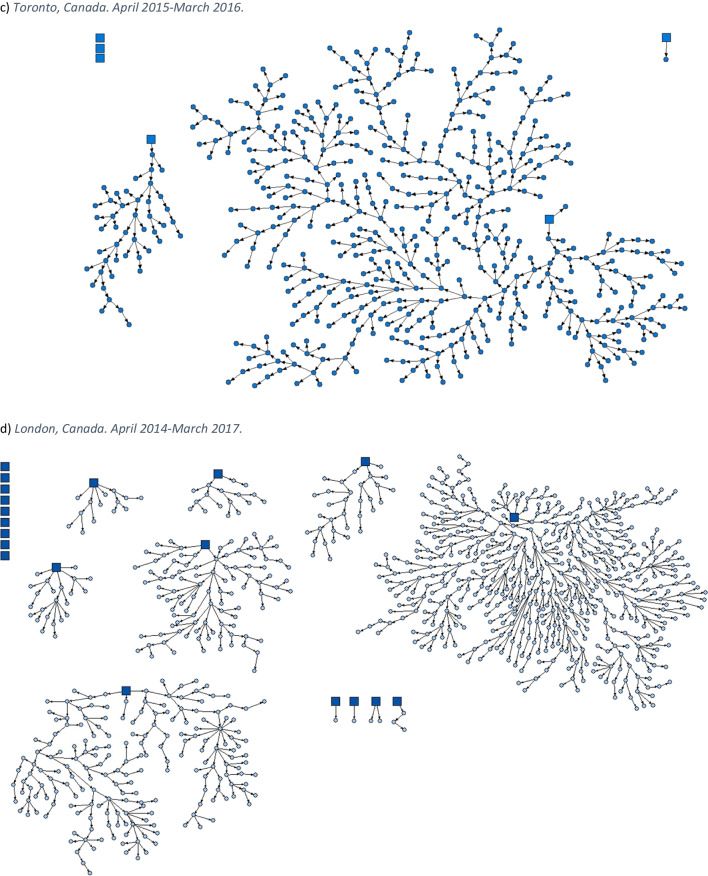

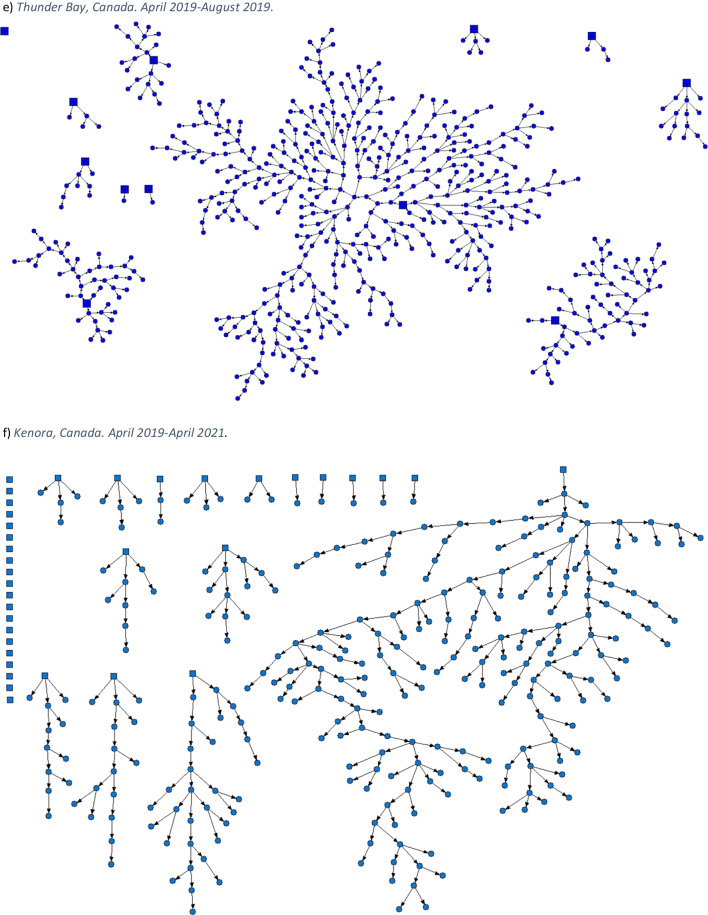
Strength of social networks and kin systems: RDS recruitment diagrams from OHC study sites. Seeds represented by squares, recruits represented by circles. **a** Hamilton, Canada. December 2009–April 2010. **b** Ottawa, Canada. January 2009–2010. **c** Toronto, Canada. April 2015–March 2016. **d** London, Canada. April 2014–March 2017. **e** Thunder Bay, Canada. April 2019–August 2019. **f** Kenora, Canada. April 2019–April 2021

**[Corrected] Fig. 1:**
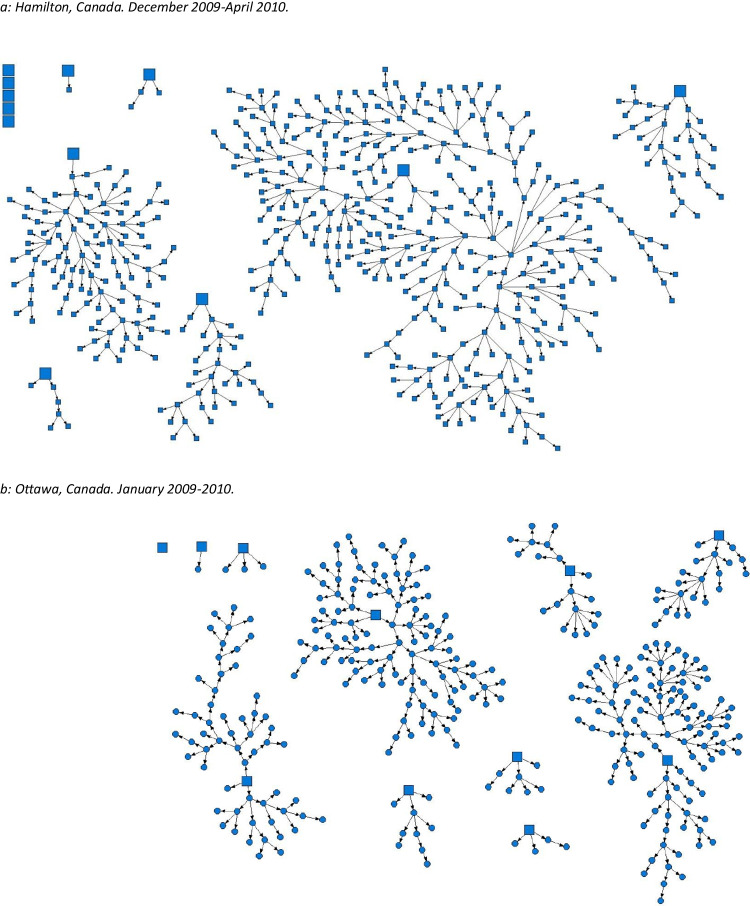

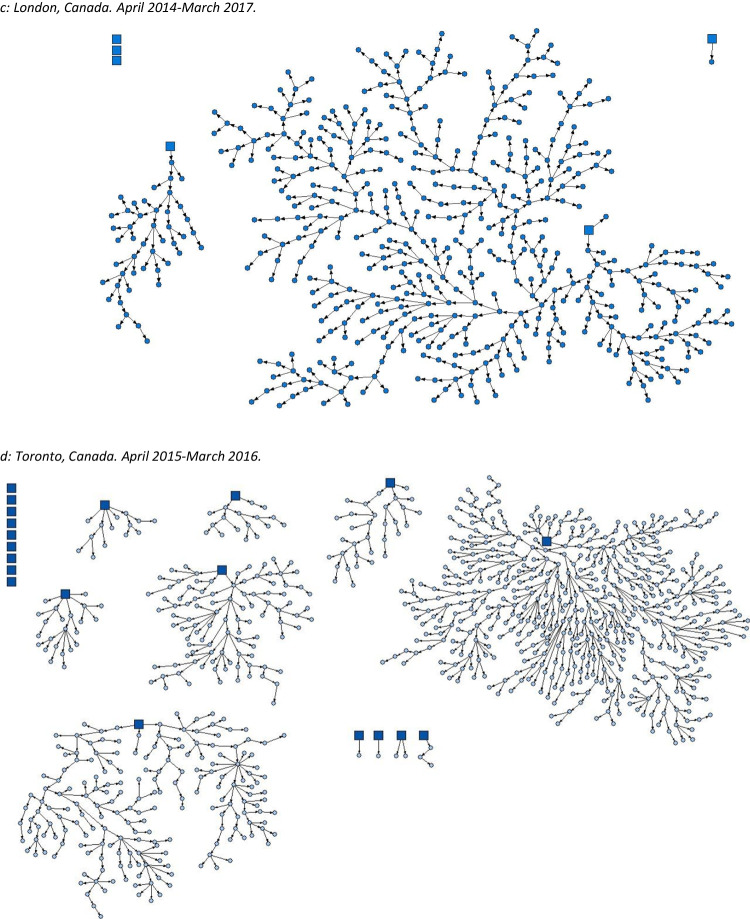

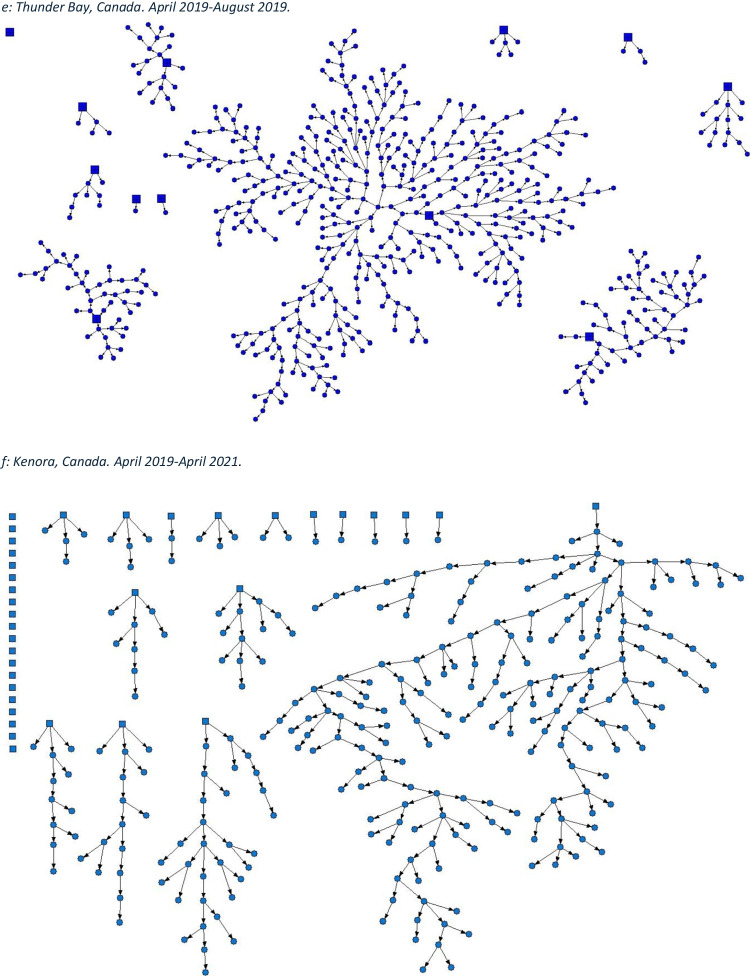
Strength of social networks and kin systems: RDS recruitment diagrams from OHC study sites. Seeds represented by squares, recruits represented by circles. **a** Hamilton, Canada. December 2009–April 2010. **b** Ottawa, Canada. January 2009–2010. **c** London, Canada. April 2014–March 2017. **d** Toronto, Canada. April 2015–March 2016. **e** Thunder Bay, Canada. April 2019–August 2019. **f** Kenora, Canada. April 2019–April 2021

